# Supplementary material for: Dopamine promotes aggression in mice via ventral tegmental area to lateral septum projections
Source: Nat Commun. 2021 Nov 23;12:6796. doi: 10.1038/s41467-021-27092-z (PMC8610979; doi:10.1038/s41467-021-27092-z)
Supplement: Supplementary file 1 — Supplementary Information [file 41467_2021_27092_MOESM1_ESM.pdf]

Supplementary Information:

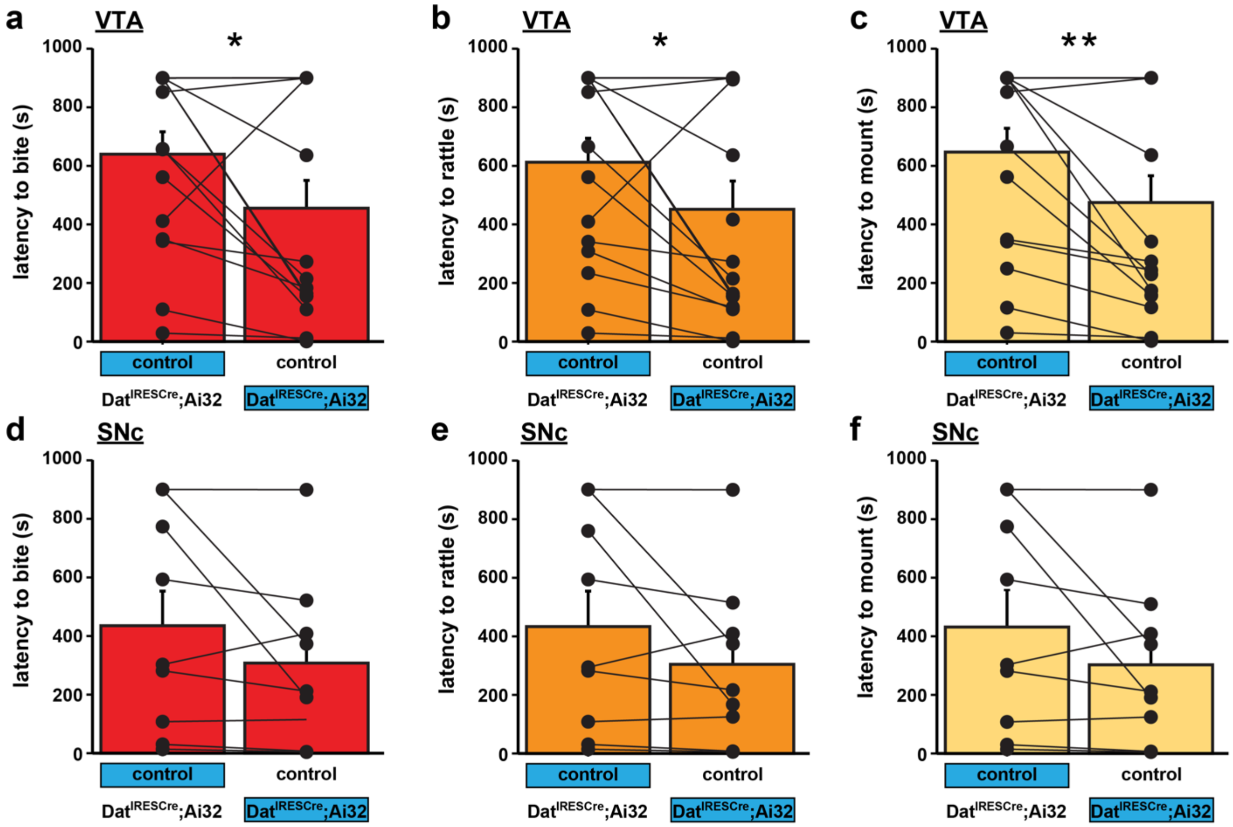

Supplementary Figure 1. Optogenetic stimulation of DAergic VTA, not SNc, neurons decrease the latency to attack. Heightened aggressive behavior, measured as decreased latency to bite (**a**), rattle (**b**), and mount (**c**) was observed in pairs when $DAT^{IRESCre}$; Ai32 mutant mice were stimulated (blue) in the VTA. (**d**-**f**) No effect of stimulation was detected during SNc stimulation. *, $P < 0.05$; **, $P < 0.01$ compared with their respective controls; mean ± SEM; $n = 16$ (**a**-**c**) and 10 (**d**-**f)** pairs.

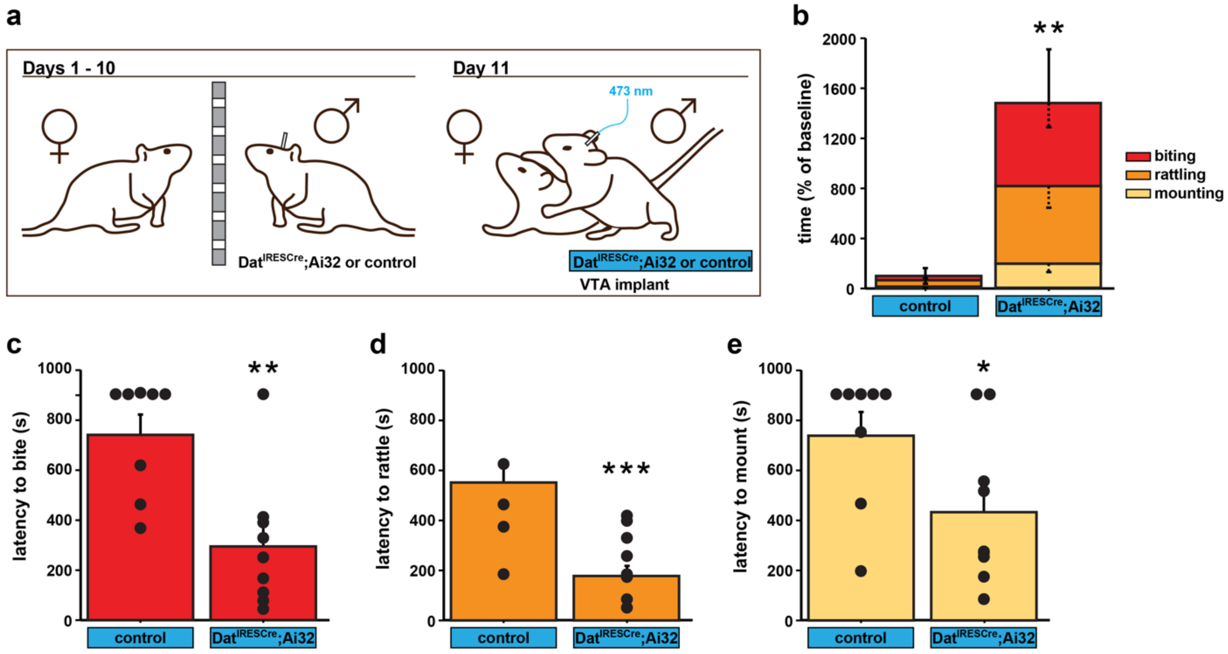

Supplementary Figure 2. ChR2-based optogenetic stimulation of DAergic VTA neurons increases aggression against females. (**a**) Schematic of the isolation-induced aggression experimental design against females. After isolation for 10 days, either $DAT^{IRESCre}$; Ai32 or control mice were stimulated (blue) in the VTA during an encounter with a female on Day 11. Heightened aggressive behavior including increased time spent fighting (**b**) and decreased latency to bite (**c**), rattle (**d**), and mount (**e**) was observed when $DAT^{IRESCre}$; Ai32 mutant mice were stimulated in the VTA in comparison to when the controls were stimulated. *, $P < 0.05$; **, $P < 0.01$; ***, $P < 0.001$ compared with their respective controls; mean ± SEM; n (control) = 8; n ($DAT^{IRESCre}$;Ai32) = 9

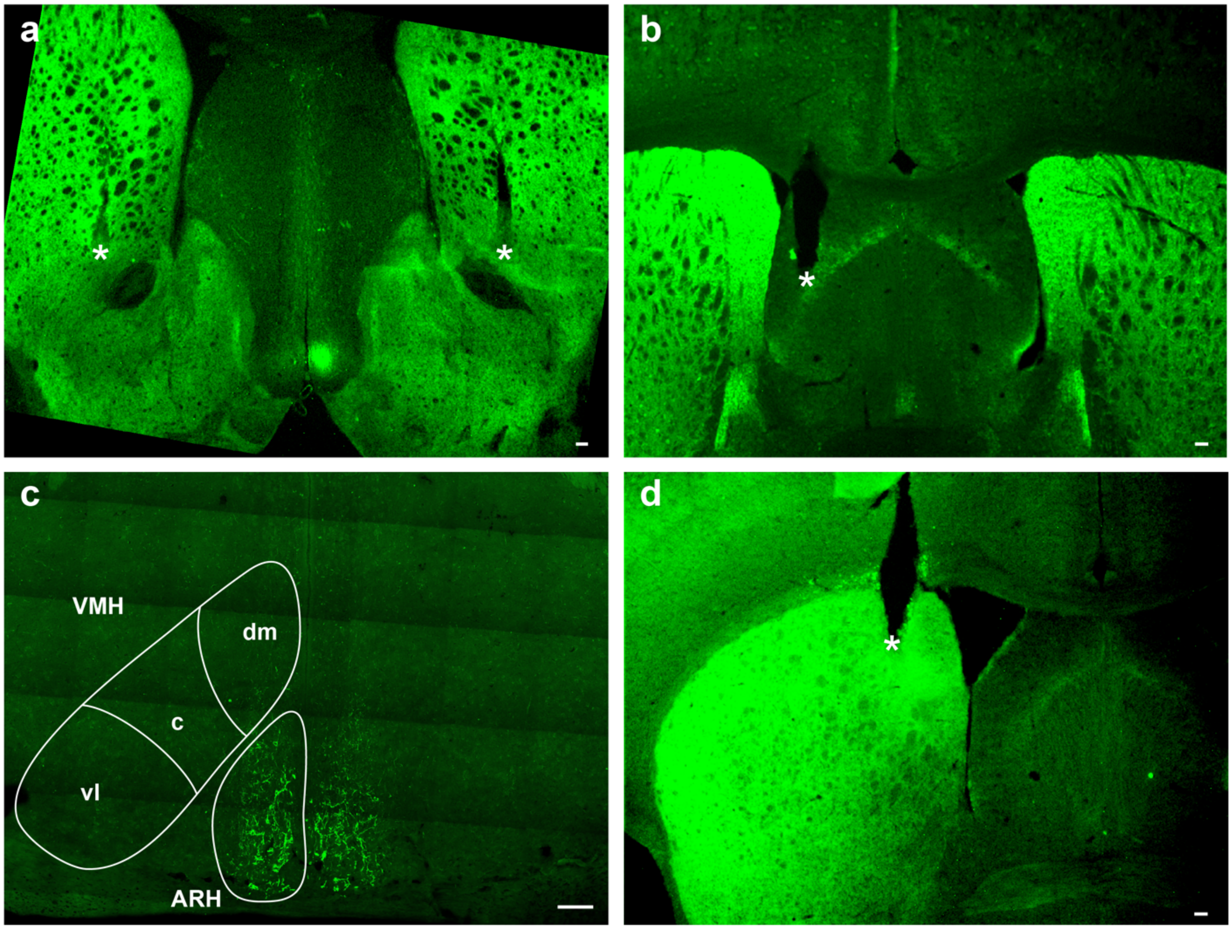

Supplementary Figure 3. DAergic innervation assessed by EYFP distribution in DAT^{IRESCre}; Ai32 mice. Histological confirmation of prominent DAergic input into the (**a**) NAc and (**b**) LS but not the (**c**) VMHvl. *Exemplary fiberoptic placement tracks in behavioral cohorts of DAT^{IRESCre}; Ai32 mutant mice expressing ChR2-eYFP in the (**a**) NAc (**b**) LS and (**d**) DS. Scale bars: 100 µm. Asterisks indicate the ventral tip of implant location.

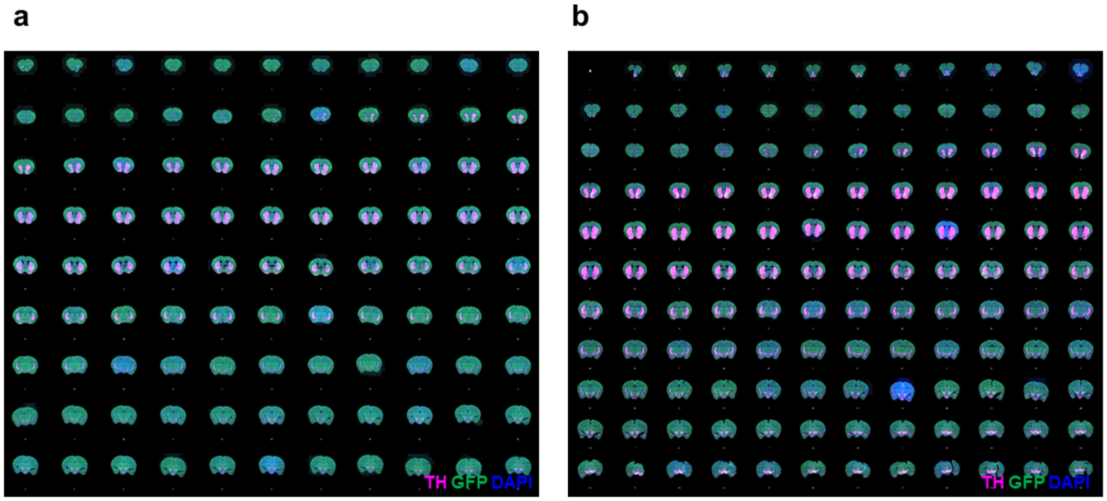

Supplementary Figure 4. Brain-wide imaging of Retro-HSV injected brains. (a) Brain-wide image montages created via Brain J[28] from the retro HSV injected into **(a)** LS and **(b)** NAc. Each section was double labeled with anti-TH (magenta), anti-GFP (green), and DAPI (blue).

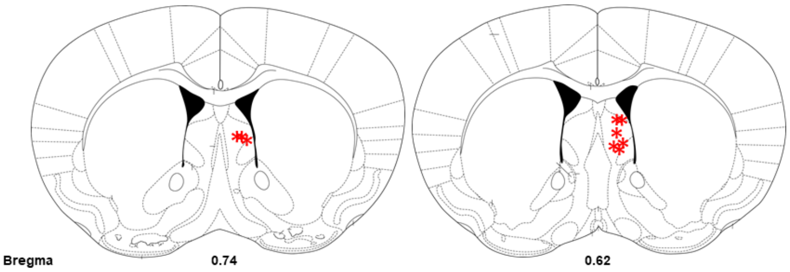

Supplementary Figure 5. Optic Fiber placements in LS for DAT^{IREScre};Ai32 activation experiment. Optic fiber location indicated by red stars from the animals used in Fig. 3a-e.

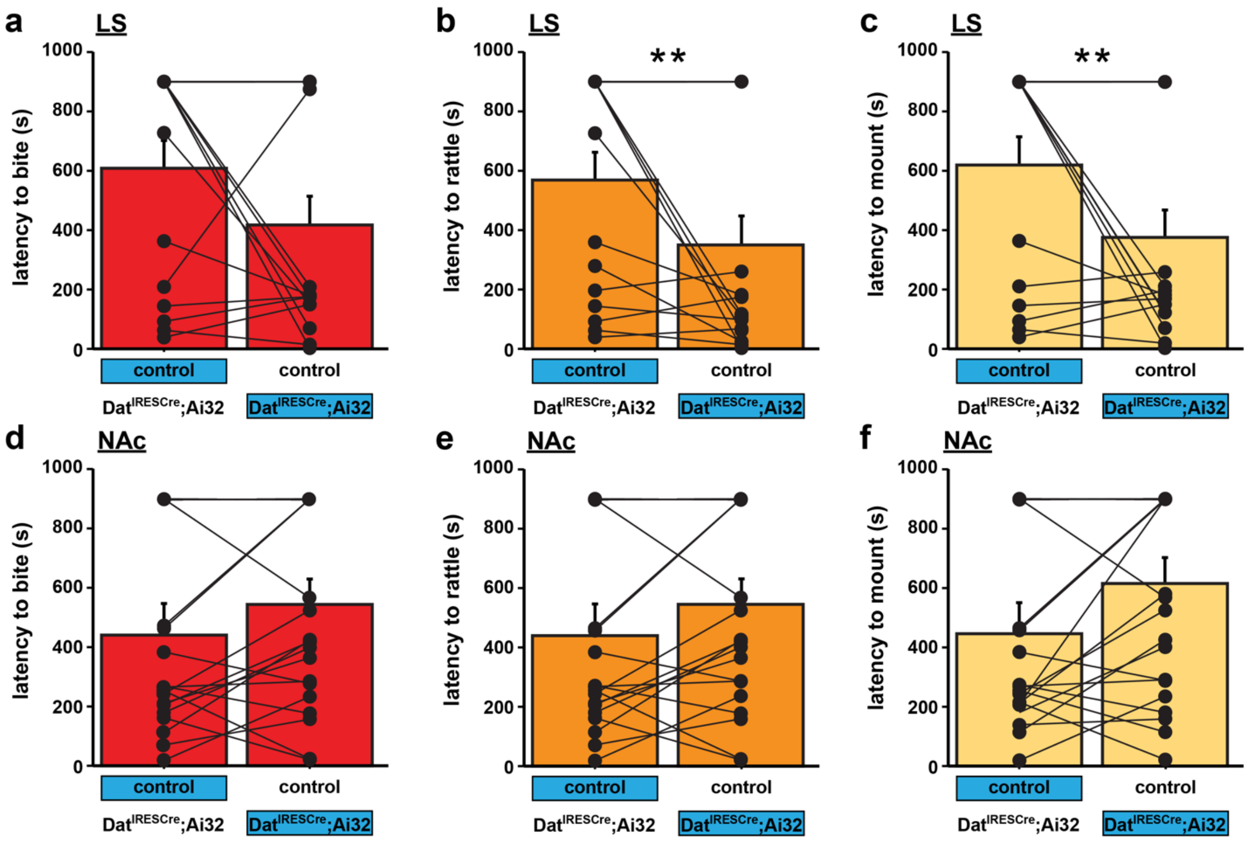

Supplementary Figure 6. DAergic projections from the VTA to the LS, not NAc, decrease the latency to attack. Heightened aggressive behavior, measured as decreased latency to bite (**a**), rattle (**b**), and mount (**c**) was observed in pairs when DAT^{IRESCre};Ai32 mutant mice were stimulated (blue) in the LS. (**d**-**f**) No effect of stimulation was detected during NAc stimulation. **, $P < 0.01$ compared with their respective controls; mean ± SEM; $n = 16$ (**a**-**c**) and 20 (**d**-**f**) pairs.

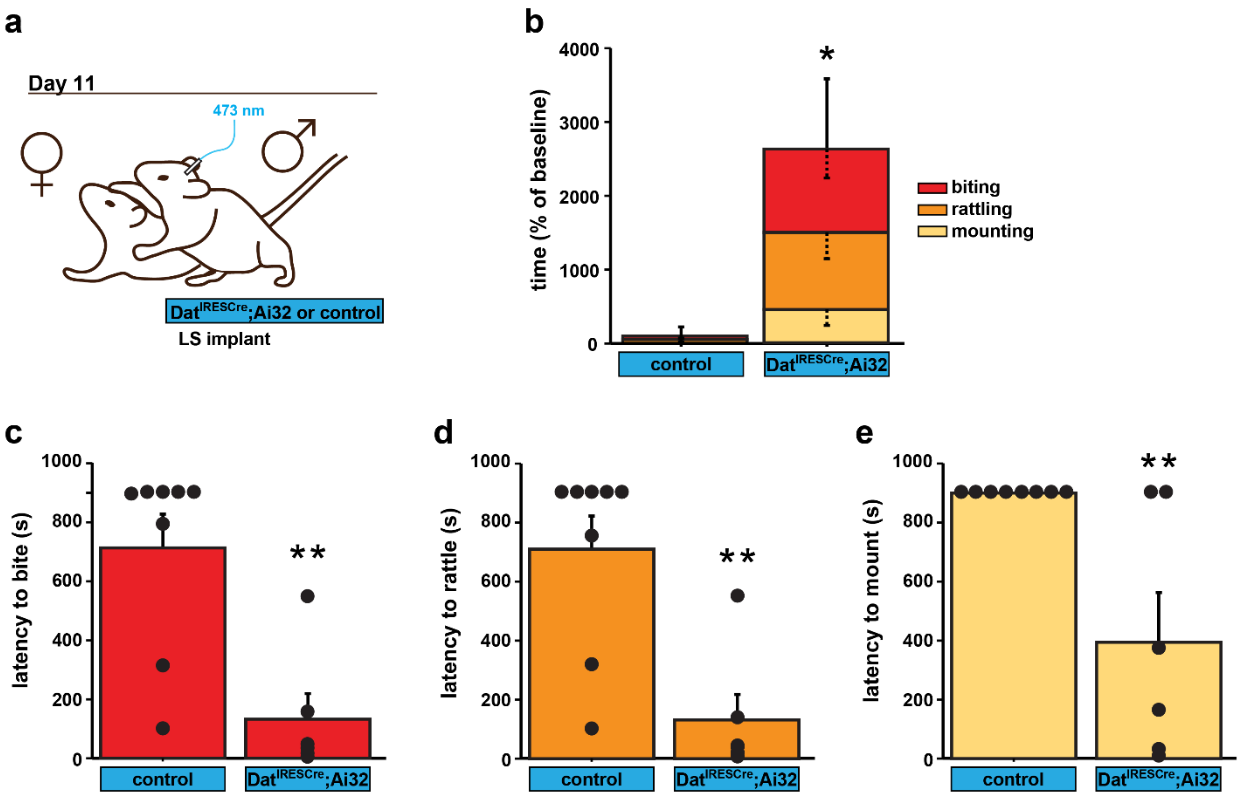

Supplementary Figure 7. ChR2-based optogenetic stimulation of DAergic projections to the LS increases aggression against females. (**a**) Schematic of DAT^{IRESCre}; Ai32 mutant or control mice being stimulated in the LS during an encounter with a female on Day 11. Heightened aggressive behavior including (**b**) increased time spent fighting and decreased latency to bite (**c**), rattle (**d**), and mount (**e**) was observed when DAT^{IRESCre}; Ai32 mutant mice were stimulated (blue) in the LS in comparison to when the controls were stimulated. *, *P* < 0.05; **, *P* < 0.01 compared with their respective controls; mean ± SEM; n (control) = 8; n (DAT^{IRESCre};Ai32) = 6.

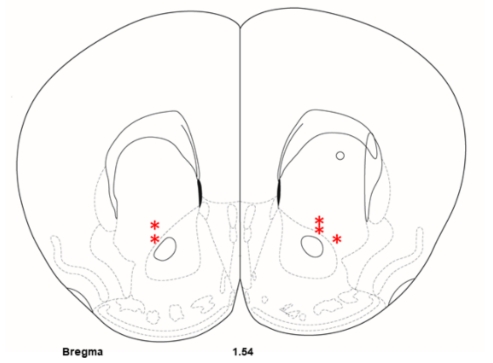

Supplementary Figure 8. Optic Fiber placements within NAc for DAT^{IREScre};Ai32 activation experiment. Optic fiber location indicated by red stars from the animals used in Fig. 3f-J.

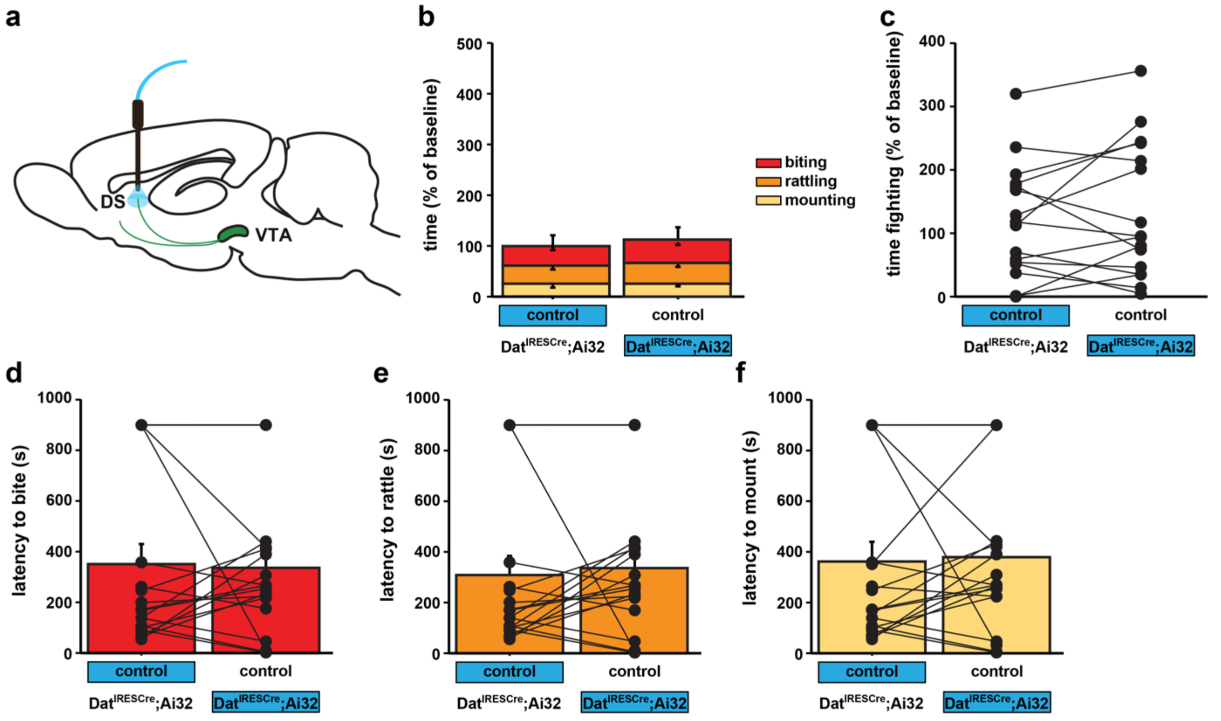

Supplementary Figure 9. ChR2-based optogenetic stimulation of off-target DAergic fibers in the DS has no effect on aggressive behavior. (**a**) The schematic diagram for stimulating DS DA release *in vivo.* No effect of stimulation (blue) was detected during DS stimulation for time spent fighting (**b, c**) and latency to bite (**d**), rattle (**e**), and mount (**f**). Mean ± SEM; *n* = 19 pairs.

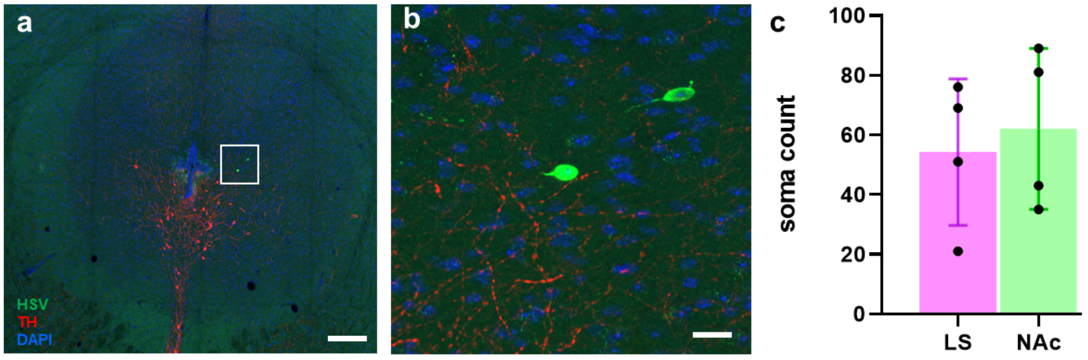

Supplementary Figure 10. Retrograde labeling of HSV virus in PAG from the LS injected brain. (**a**) HSV infected cell body in the PAG (scale bar: 200 µm). (**b**) Higher magnification image of the HSV infected cells (scale bar: 20 µm); anti-TH (Red), anti-GFP (green), and DAPI (blue). (**c**) Quantification of soma in PAG of LS and NAc injected brain (n = 4), data are presented Mean ± SEM.

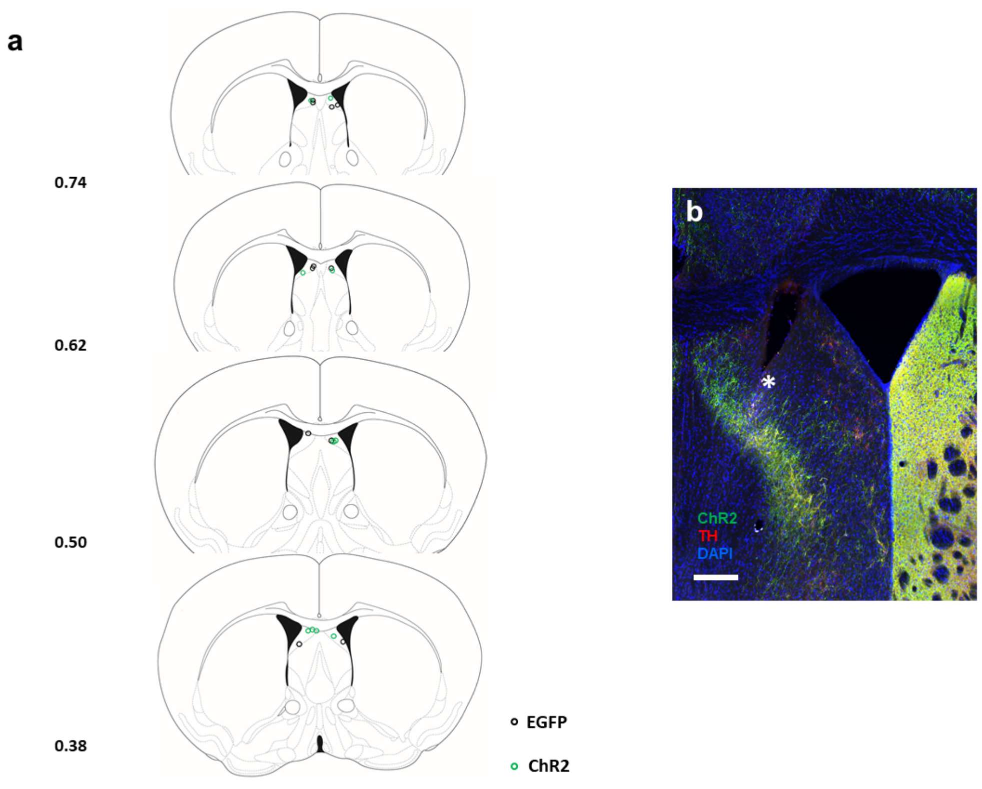

Supplementary Figure 11. Optic Fiber placements for viral ChR2 activation experiment. (**a**) Optic fiber location indicated by black (EGFP) and green (ChR2) circles from the animals used in Fig. 4. (**b**) Representative image of optical fiber location from the ChR2 injected animal; scale bar: 200 µm, TH (Red), ChR2 (green) and DAPI (blue). Asterisks indicate the ventral tip of implant location.

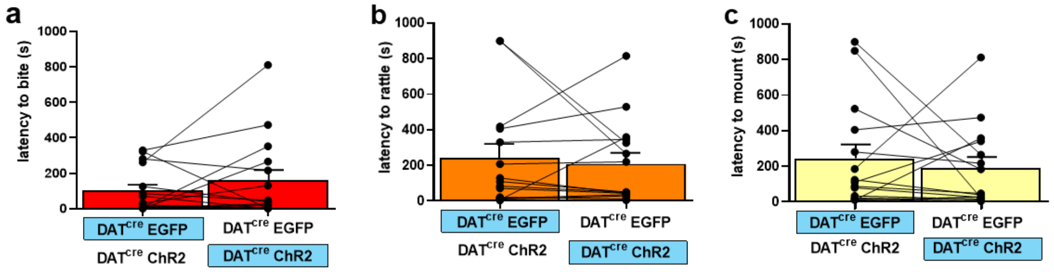

Supplementary Figure 12. Optogenetic activation of VTA-LS DAergic fibers does not alter latency to attack. The latency to bite (**a**), rattle (**b**), and mount (**c**) was not altered in pairs when $DAT^{IRESCre}$ ChR2 injected mice were stimulated (blue) in the LS. n= 15 pairs (a), (b), and (c), data are presented Mean ± SEM.

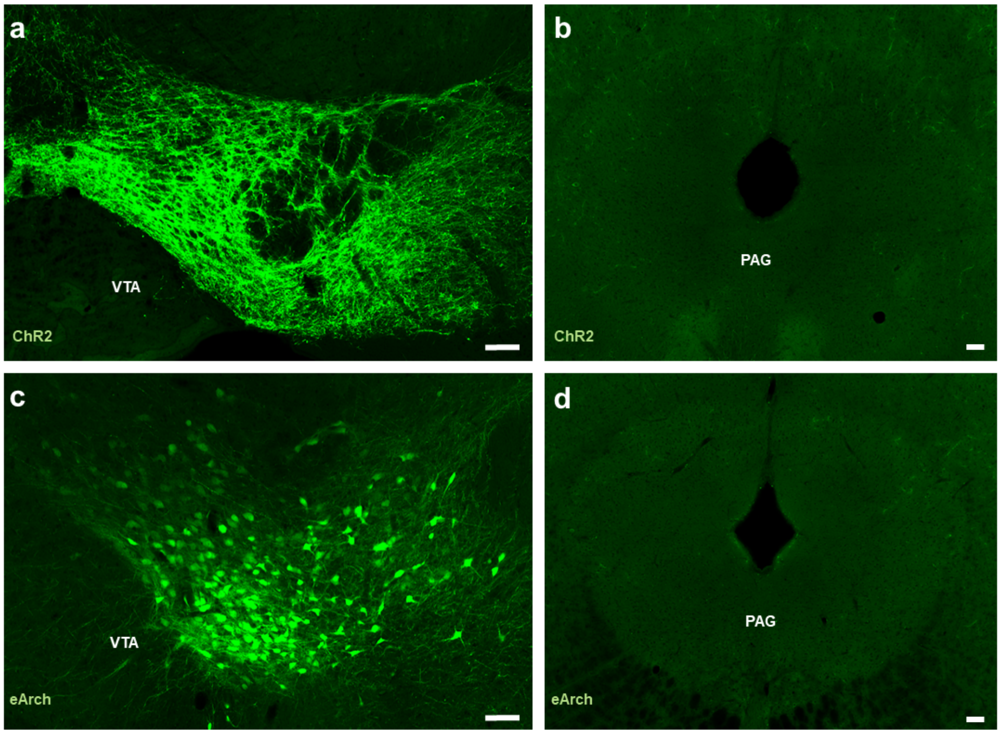

Supplementary Figure 13. Periaqueductal gray image from ChR2 injected animal. (a) ChR2 expression in VTA, ChR2 (green) scale bar: 100 µm **(b)** No ChR2 infected cell body was found in PAG from the animals used in Fig. 4; ChR2 (green) scale bar: 100 µm.(**c**) eArch expression in VTA, eArch (green) scale bar: 100 µm (**d**) No ChR2 infected cell body was found in PAG from the animals used in Fig. 6; eArch (green), scale bar: 100 µm.

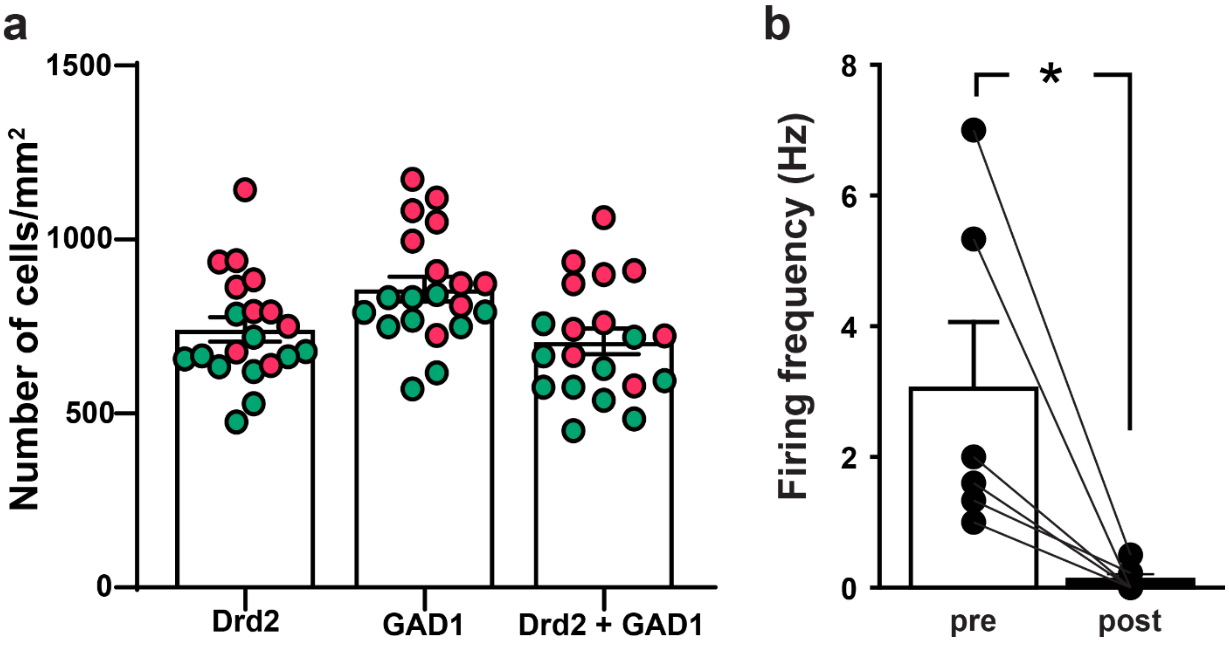

Supplementary Figure 14. RNAscope analysis for *GAD1* and *Drd2* mRNA colocalization in the LS and firing frequency of LS neurons with IPSP responses. (a) Brain sections containing the LS were stained for Drd2 and GAD1. Drd2, GAD1 and Drd2+GAD1 positive cells were counted in the outlined LS. The number of cells per area was calculated and plotted. (N = 20 sections, 2 mice – color indicated), data are presented Mean ± SEM. **(b)** Average firing frequency of LS neurons that respond to blue light stimulation with an IPSP during 0.5 s window before train stimulation (pre) and during and after train stimulation (post). n of recorded cells = 6, n of recorded animals = 5,

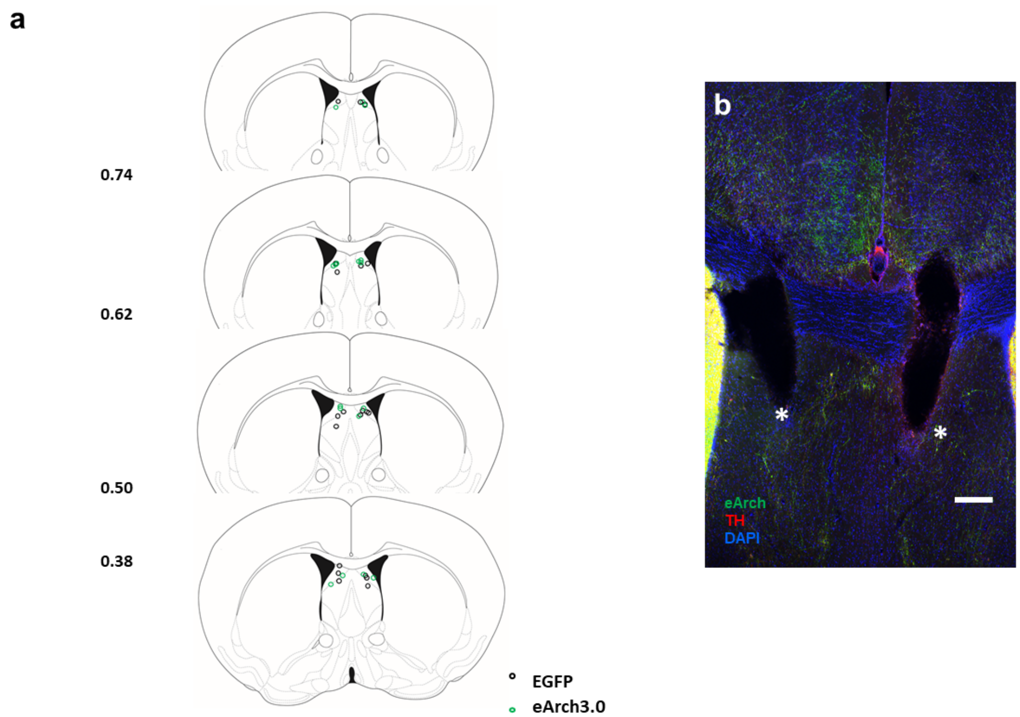

Supplementary Figure 15. Optic Fiber placements for eArch inhibition experiment. (**a**) Optic fiber location indicated by black (EGFP) and green (eArch) circles from the animals used in Fig. 6. (**b**) Representative image of optical fiber location from the eArch injected animal; scale bar: 200 µm, TH (Red), ChR2 (green) and DAPI (blue). Asterisks indicate the ventral tip of implant location.

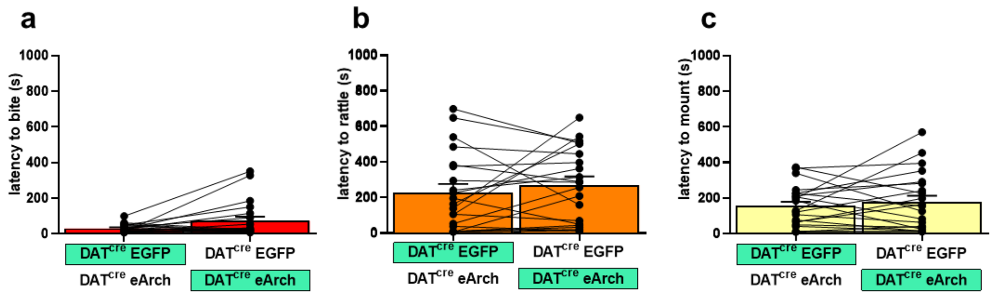

Supplementary Figure 16. Optogenetic inhibition of VTA-LS DAergic fibers does not alter latency to attack. The latency to bite (**a**), rattle (**b**), and mount (**c**) was not altered in pairs when DAT^{IRESCre} eArch injected mice were stimulated (green) in the LS. n= 21 pairs (a), (b), and (c), data are presented Mean ± SEM.

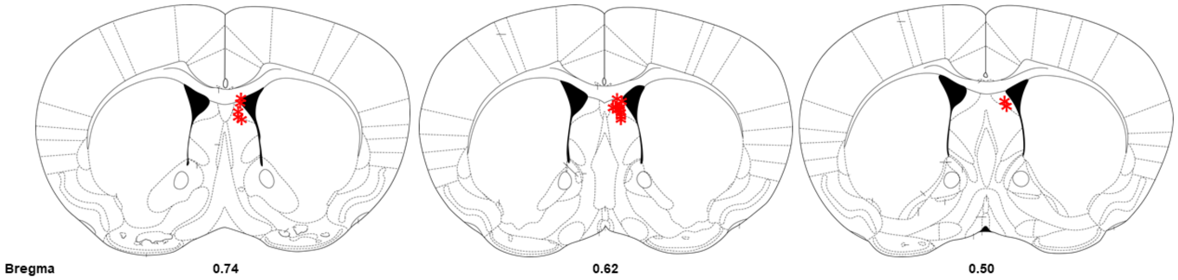

Supplementary Figure 17. Cannula placements within LS for sulpiride infusion experiment. (a) Cannula location indicated by red stars from the animals used in Fig. 7a-g.

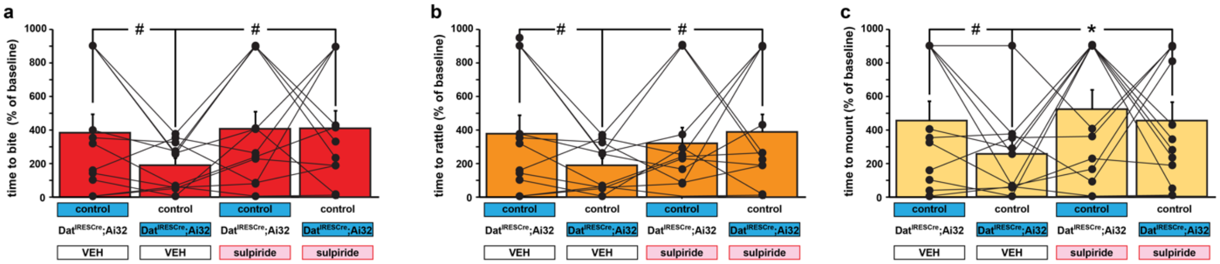

Supplementary Figure 18. Terminal release of DA in the LS is necessary for VTA DAergic activity to decrease the latency to attack. Pharmacological rescue of the latency to bite (**a**), rattle (**b**), and mount (**c**) through local D2 receptor antagonism in the LS. $^{\#}$, $P < 0.1$, *, $P < 0.05$compared with their respective controls; mean ± SEM; n = 11 pairs. VEH, vehicle.
