# Supplementary material for: Dopamine promotes aggression in mice via ventral tegmental area to lateral septum projections
Source: Nat Commun. 2021 Nov 23;12:6796. doi: 10.1038/s41467-021-27092-z (PMC8610979; doi:10.1038/s41467-021-27092-z)
Supplement: Supplementary file 3 — Description of Additional Supplementary Files [file 41467_2021_27092_MOESM3_ESM.pdf]

Description of Additional Supplementary Files

File Name: Supplementary Data 1
Description: Statistical analyses.

File Name: Supplementary Movie 1
Description: **3D render of retrogradely labelled neurons in VTA and PAG.** Brain-wide 3D animation of segmented VTA and PAG somas from one LS-injected mouse (magenta) and one NAc-injected mouse (green). The VTA and PAG appear in grey during the rotation.
