# Supplementary material for: Dopamine promotes aggression in mice via ventral tegmental area to lateral septum projections
Source: Nat Commun. 2021 Nov 23;12:6796. doi: 10.1038/s41467-021-27092-z (PMC8610979; doi:10.1038/s41467-021-27092-z)
Supplement: Supplementary file 6 — Reporting Summary [file 41467_2021_27092_MOESM6_ESM.pdf]

nature research

Corresponding author(s): Mark Ansorge

Last updated by author(s): 2021/05/26

Reporting Summary

Nature Research wishes to improve the reproducibility of the work that we publish. This form provides structure for consistency and transparency in reporting. For further information on Nature Research policies, see our Editorial Policies and the Editorial Policy Checklist.

Statistics

For all statistical analyses, confirm that the following items are present in the figure legend, table legend, main text, or Methods section.

n/a	Confirmed	
☐	☒	The exact sample size (n) for each experimental group/condition, given as a discrete number and unit of measurement
☐	☒	A statement on whether measurements were taken from distinct samples or whether the same sample was measured repeatedly
☐	☒	The statistical test(s) used AND whether they are one- or two-sided<br>*Only common tests should be described solely by name; describe more complex techniques in the Methods section.*
☐	☒	A description of all covariates tested
☐	☒	A description of any assumptions or corrections, such as tests of normality and adjustment for multiple comparisons
☐	☒	A full description of the statistical parameters including central tendency (e.g. means) or other basic estimates (e.g. regression coefficient) AND variation (e.g. standard deviation) or associated estimates of uncertainty (e.g. confidence intervals)
☐	☒	For null hypothesis testing, the test statistic (e.g. F, t, r) with confidence intervals, effect sizes, degrees of freedom and P value noted<br>*Give P values as exact values whenever suitable.*
☒	☐	For Bayesian analysis, information on the choice of priors and Markov chain Monte Carlo settings
☒	☐	For hierarchical and complex designs, identification of the appropriate level for tests and full reporting of outcomes
☒	☐	Estimates of effect sizes (e.g. Cohen's d, Pearson's r), indicating how they were calculated

Our web collection on statistics for biologists contains articles on many of the points above.

Software and code

Policy information about availability of computer code

Data collection	Behavioral Data were collected using ANY-maze (version 6.23) video tracking software (Stoelting Co.). Electrophysiological data were collected using pClamp 10 (Molecular Devices). Whole brain images were acquired using slide scanner AZ100 microscope equipped with a 4x 0.4NA Plan Apo objective (Nikon Instruments Inc) and P200 slide loader (Prior Scientific), controlled by NIS-Elements using custom acquisition scripts (Nikon Instruments Inc.). Confocal images were taken using a laser confocal scanning microscope (Leica, TCS SP8).
Data analysis	Electrophysiological data were analyzed with Axograph X (Axograph Scientific). Analysis of brain wide images were performed using WholeBrain Software (Furth et al., 2018). RNAscope data were analysed by LASx software (Leica, TCS SP8). Statistical analysis were done using StatView 5.0 software (SAS Institute, Cary, NC, USA) or Prism9 software (GraphPad) and Microsoft Excel.

For manuscripts utilizing custom algorithms or software that are central to the research but not yet described in published literature, software must be made available to editors and reviewers. We strongly encourage code deposition in a community repository (e.g. GitHub). See the Nature Research guidelines for submitting code & software for further information.

Data

Policy information about availability of data

All manuscripts must include a data availability statement. This statement should provide the following information, where applicable:

- Accession codes, unique identifiers, or web links for publicly available datasets
- A list of figures that have associated raw data
- A description of any restrictions on data availability

The data supporting the findings of this study are available in the paper and Supplementary Information files or from the corresponding author upon reasonable request.

Field-specific reporting

Please select the one below that is the best fit for your research. If you are not sure, read the appropriate sections before making your selection.

☒ Life sciences ☐ Behavioural & social sciences ☐ Ecological, evolutionary & environmental sciences

For a reference copy of the document with all sections, see nature.com/documents/nr-reporting-summary-flat.pdf

Life sciences study design

All studies must disclose on these points even when the disclosure is negative.

Sample size	No statistical analysis were done to determine the sample size. Using preliminary data and results from previous study from our group (Yu et al. 2014) we decides the sample size. Sample sizes are indicated in the legends of Figures and Supplementary Figures.
Data exclusions	No data was excluded from the study
Replication	In some instances, small pilot studies were performed to establish experimental conditions and thus were then replicated by the subsequent experiments which are presented here. Furthermore, data in Fig 1b -d is a replication of data in Yu et al., 2014., serving as a positive control for the negative finding in Fig1e-g and demonstrating specificity. Rest of the figures are not replication of any other experiments.
Randomization	Mice were always randomly assigned to the behavioral groups.
Blinding	The investigator was blinded during group allocation, data collection and analysis for all the behavioral experiment. Electrophysiologial experiment blinding was not necessary as all the experiments were performed under same condition.

Reporting for specific materials, systems and methods

We require information from authors about some types of materials, experimental systems and methods used in many studies. Here, indicate whether each material, system or method listed is relevant to your study. If you are not sure if a list item applies to your research, read the appropriate section before selecting a response.

Materials & experimental systems

n/a	Involved in the study	
☐	☒	Antibodies
☒	☐	Eukaryotic cell lines
☒	☐	Palaeontology and archaeology
☐	☒	Animals and other organisms
☒	☐	Human research participants
☒	☐	Clinical data
☐	☐	Dual use research of concern

Methods

n/a	Involved in the study	
☒	☐	ChIP-seq
☒	☐	Flow cytometry
☒	☐	MRI-based neuroimaging

Antibodies

Antibodies used	*Describe all antibodies used in the study; as applicable, provide supplier name, catalog number, clone name, and lot number.*
Validation	*Describe the validation of each primary antibody for the species and application, noting any validation statements on the manufacturer's website, relevant citations, antibody profiles in online databases, or data provided in the manuscript.*

Animals and other organisms

Policy information about studies involving animals; ARRIVE guidelines recommended for reporting animal research

Laboratory animals	DATIRESCre (Backman et al., 2006) and Ai32 (RCL-ChR2(H134R)/EYFP) (Madisen et al., 2012) mice were crossed to produce experimental cohorts consisting of DATIRESCre; Ai32 and single mutant controls on a mixed F1 background (C57BL/6J x 129S2/129SvEv/Tac).<br>Either DATIREScre or DATIREScre;Ai32 were used in the experiments.<br>For the RNAscope experiment, Drd2-EGFP, GENSAT - S118Gsat/Mmnc mice were used.<br>2 months old male mice were used in the experiments.<br>Extended Fig.2 & 7: 2 months old DATIREScre;Ai32 female mice were used.
Wild animals	No wild animals were used.
Field-collected samples	No field collected sample was used.

Ethics oversight	All animal testing was conducted under protocols approved by Columbia University and New York State Psychiatric Institute Institutional Animal Care and Use Committees.

Note that full information on the approval of the study protocol must also be provided in the manuscript.

Dual use research of concern

Policy information about dual use research of concern

Hazards

Could the accidental, deliberate or reckless misuse of agents or technologies generated in the work, or the application of information presented in the manuscript, pose a threat to:

No	Yes	
☐	☐	Public health
☐	☐	National security
☐	☐	Crops and/or livestock
☐	☐	Ecosystems
☐	☐	Any other significant area

Experiments of concern

Does the work involve any of these experiments of concern:

No	Yes	
☐	☐	Demonstrate how to render a vaccine ineffective
☐	☐	Confer resistance to therapeutically useful antibiotics or antiviral agents
☐	☐	Enhance the virulence of a pathogen or render a nonpathogen virulent
☐	☐	Increase transmissibility of a pathogen
☐	☐	Alter the host range of a pathogen
☐	☐	Enable evasion of diagnostic/detection modalities
☐	☐	Enable the weaponization of a biological agent or toxin
☐	☐	Any other potentially harmful combination of experiments and agents
